# Taking Open Innovation to the Molecular Level - Strengths and Limitations

**DOI:** 10.1002/minf.201200014

**Published:** 2012-08-07

**Authors:** Barbara Zdrazil, Niklas Blomberg, Gerhard F Ecker

**Affiliations:** a University of Vienna, Department of Medicinal Chemistry, Pharmacoinformatics Research GroupAlthanstrasse 14, 1090 Vienna, Austria; b Medicinal Chemistry, Respiratory and Inflammation iMEDAstraZeneca R&D Mölndal, S-43183 Mölndal, Sweden

**Keywords:** Open Innovation, Open access, Open PHACTS, Data retrieval, Bioassay ontology

## Abstract

The ever-growing availability of large-scale open data and its maturation is having a significant impact on industrial drug-discovery, as well as on academic and non-profit research. As industry is changing to an ‘open innovation’ business concept, precompetitive initiatives and strong public-private partnerships including academic research cooperation partners are gaining more and more importance. Now, the bioinformatics and cheminformatics communities are seeking for web tools which allow the integration of this large volume of life science datasets available in the public domain. Such a data exploitation tool would ideally be able to answer complex biological questions by formulating only one search query. In this short review/perspective, we outline the use of semantic web approaches for data and knowledge integration. Further, we discuss strengths and current limitations of public available data retrieval tools and integrated platforms.

## 1 Introduction

In the digital age, concepts like ‘open source’ and ‘open access’ are gaining more and more attentiveness. Owning information or data does no longer guarantee commercial success. It is the way to use these data and to combine them with available knowledge that makes the difference and provides a competitive edge.^1^,^2^ In this context, a business concept which tries to maximize data exploitation by creating and making use of synergies between internal and external knowledge is most promising. This concept, termed ‘open innovation’, is one strategy by which pharmaceutical industry may overcome the current crisis caused by declining productivity, patent expiries and a downward trend in drug pricing by acknowledging and actively involving the creative capacity outside the traditional pharmaceutical R&D units. In parallel, with the growth of academic drug discovery research, translational biology and biological data explosion, the data analysis activities in industry are shifting to benefit from the high-quality, open and accessible data out in the world wide web.^3^,^4^

It is obvious that also *academia* benefits from the maturation of the public-domain and from strong public-private partnerships with industry. For example, nowadays researchers have the ability to access patent databases as well as databases of clinical candidates. This paves the way for repurposing of drugs for activity in unexpected indications^5^ and fosters efforts from non-profit organizations towards rare diseases.^6^,^7^

The ‘democratization’^8^ of data, where academic teams can access large scale resources previously only accessible inside large organisations, gives an unprecedented opportunity to tackle difficult research problems related to human health. There remain many diseases with poorly met medical need and where fundamental understanding of disease mechanisms is needed. Similarly, in the field of medicinal chemistry there are many areas where an investment in basic research is required to understand how we effectively target the protein-protein interactions fundamental to cellular regulation,^9^ enzyme regulation (e.g. AMP kinase activators),^10^ or modulating highly complex cellular networks.^11^ Similarly, the increasing applicability of high content biology and multiplexed assays coupled with systems analysis and molecular intervention^12^ offers new opportunities for medicinal chemistry to change and possibly reverse disease processes.

However, data organization, integration and management are far from being trivial. Proper usage for drug discovery can only be assured by a thorough investigation of data quality and integrity. Herein, we want to draw the attention to the opportunities offered by semantic web technologies for flexible integration of databases and we are discussing strengths and weaknesses of open innovation when it comes to the molecular level, exemplified by a case study on ABC transporters and respective assays.

## 2 A New Era of Open Innovation

### 2.1 Data Sources for Drug Discovery and Design

Traditionally, pharmaceutical companies were relying on their *in house* repositories of compound bioactivity data for the purpose of finding new lead candidates in the drug discovery process. Historically, the pharmaceutical industry and related academic fields sought to protect and confine ideas and data inside the organisation. Such ‘intellectual mercantilism’ or ‘closed innovation’ is driven by the motivation to prevent your intellectual property being exploited by rivals.

During the last decade, however, things have changed fundamentally, and much as free trade has fuelled growth and fundamentally changed patterns of economic activity, we are now experiencing increased scientific collaboration between *academia*, industry and governmental institutions. The increase in external research, innovation scouting (e.g. Eli Lilly’s Innocentive) and public private partnerships is driving new, open, models of drug discovery research. An important driver for this change has been large scale initiatives such as the NIH Molecular Libraries programme. As a consequence, an unprecedented body of data on compound bioactivities has been entering the public domain. For instance, the PubChem BioAssay database currently contains 500 000 descriptions of assay protocols, covering 5000 protein targets, 30 000 gene targets and providing over 130 million bioactivity outcomes. It hosts mainly screening data generated by the NIH Molecular Libraries and Imaging Program (MLP; http://commonfund.nih.gov/molecularlibraries/).^13^

ChemBank is a small-molecule database which stores screening data coming from the Broad Institute of Harvard and MIT,^14^ and the DrugBank database contains more than 4100 drug entries including FDA approved small molecule and biotech drugs as well as 3200 experimental drugs.^15^

Complementary to these efforts, in 2010 the ChEMBL database (ChEMBLdb) was transferred from the private into the public sector. Being funded for five years, ChEMBLdb and related tools are being developed by the group of J. Overington at the European Bioinformatics Institute. In contrast to others, data in there are manually curated, and thus of higher quality. However, data from patents is so far not included.^16^,^17^

One should also not forget about the more specialized data sources serving the needs of specific research communities, such as the transporter database TPsearch (http://125.206.112.67/tp-search/login.php), or the IUPHAR database containing data from G-Protein-Coupled Receptors, Voltage-Gated Ion Channels, Ligand-Gated Ion Channels and Nuclear Hormone Receptors (http://www.iuphar-db.org/).^18^

The ever pressing need to organize, catalogue and rate these data resources, so that the information they contain can be most effectively exploited, is reflected by the existence of a special wiki for databases termed MetaBase (MB) (http://metadatabase.org/wiki/Main_Page). It is a community-curated database containing more than 2000 commonly used biological databases.^19^ Even more Nucleic Acids Research releases every year a special database issue, recently featuring descriptions of 92 new online databases covering various areas of molecular biology and 100 papers describing recent updates to the databases previously described.^20^

The ever increasing availability of large-scale open data and most notably its maturation – which refers to the existence of stable and reliable platforms as well as clear terms of usage – is having a significant impact on industrial drug-discovery, as well as on academic and non-profit research. Moreover, as industry is changing to an ‘open innovation’ business concept, pre-competitive initiatives including academic research cooperation partners, such as the Innovative Medicines Initative (IMI; http://www.imi.europa.eu/), are gaining more and more importance. Of course, the question of which part of the drug discovery process is pre-competitive, always depends on the project and contributing scientists and lawyers. In general, it includes information that can be shared without conferring a commercial advantage.^3^

There is a great chance also for *academia* to benefit from these developments. *In silico* models built upon such huge data sources span a much larger chemical space than the models traditionally built using academic *in house* databases.^21^ This makes it much easier to build up reliable models and it facilitates data mining. In addition, it will encourage the development of novel tools and predictive algorithms within the public domain which will further academic as well as industry-based drug design.^17^

A classical use case for this is the depositing of more than 13 500 compound structures of possible drugs against malaria by GlaxoSmithKline (GSK) into the public domain in May 2010.^22^ It is called the Tres Cantos Antimalarial (TCAMS) dataset, and full compound assay data and structures are available at ChEMBL – Neglected Tropical Disease Archive (ChEMBL-NTD; http://www.ebi.ac.uk/chemblntd). This further led to the identification of 47 putative starting points for lead optimization in the search for new antimalarial drugs.^23^

### 2.2 Integration of Databases

Due to public data freely available and strong public-private partnerships, suddenly all scientists involved in a project have (more or less) the same sort of information available. However, the outcome of a data search still will never be the same. It depends on the type of database(s) and the search queries used in the data retrieval process, as well as on the knowledge and scientific background of the persons involved.

Diversification of results by more than one person working on a project can never be avoided completely, and it also might give valuable input to the others. However, the preparatory parts of the work process which deal with logistics such as updating, cleaning and connecting different data sources (often across multiple domains) can be optimized not only, avoiding duplication of effort but, perhaps more importantly, offers an opportunity to increase consistence and transparency of these critical steps. In the increasingly complex and sophisticated scientific data-environments this is a major challenge and significant cost.

Data integration is the promising answer to this task. Semantic Web Technology (SWT) makes it possible to integrate and search the large volume of life science datasets in the public domain^24^ and it is able to provide data in computer-readable formats – which is needed in today’s data intensive science.^25^,^26^ By using the tools such as Resource Description Framework (RDF) metadata model, which describes relationships in the form of subject-predicate-object expressions (or so called ‘triples’), statements about data resources are made. This allows building up effective connections between such data sources. A prerequisite, however, is the assignment of unique identifiers to entities in the databases (e.g. compounds) and also to concepts (e.g. “binds”, “hydrolyses” or “antagonize”).^21^

There are some prominent data projects which are using a semantic web approach for data (or knowledge) integration. First of all, there is Linked Life Data (LLD; http://linkedlifedata.com/) – an aggregation of more than 25 popular biomedical data sources. Second, Bio2RDF (http://bio2rdf.org/)^27^ integrates publicly available data from some of the most popular databases in bioinformatics. Last but not least, Chem2Bio2RDF^28^ is a prominent example of data-linkage across domains and has proven useful in specific examples of polypharmacology, multiple pathway inhibition and adverse drug reaction-pathway mapping. It integrates six categories of data based on the nature of biological/chemical concepts and their relationships, such as chemical & drug, or protein & gene. A potential draw-back is the lack of a formal ontology which increases the complexity of some queries.^24^

As a consequence, the bio- and cheminformatics communities are now seeking for data exploitation tools which allow the user to retrieve the required data easily, quickly, correctly (meaning of high-quality), and, of course, this system should be freely available and open to everyone. Additionally, such tools should be able to combine data/information in a way that one complex research query gives the desired answer(s). The ideal system should be able to answer research questions like: “Give me all compounds which have been associated with liver toxicity and list their interaction profiles with the transporters expressed in the liver”. By that, the researcher would be able to spend his time on using his/her real special expertise to foster drug-discovery output, and not being occupied with mere data retrieval, cleaning, duplicate filtering, and combination. Nevertheless, those complex web approaches are still in its childhood.

Recently, there has been a call of the Innovative Medicines Initiative (IMI) for the development of an Open Pharmacological Space (OPS), an open innovative platform for knowledge discovery and verification, freely accessible for the drug-discovery community. The winning consortium ‘Open PHACTS’ (the Open Pharmacological Concepts Triple Store; http://www.openphacts.org/) is a partnership between the European Community and the European Federation of Pharmaceutical Industries and Associations (EFPIA) and started to work in March 2011. The consortium comprises 14 European academic and SME (Small and Medium Enterprises) partners and 8 EFPIA members. The main goal is the alignment and multiscale integration of proprietary and public data sources into a single system by the use of semantic triples. It should include e.g. data on small molecules, their pharmacological profiles, pharmacokinetics, ADMET data, biological targets and pathways. It is important to mention that the Open PHACTS web service will be tailored to the particular needs of the drug-discovery community. So called ‘research questions’ or ‘business questions’ are being defined by the consortium members (either being from *academia* or EFPIA members), prioritized, and analysed. This forms the basis for deciding on which databases, software, and web tools are being integrated in the final release of the platform. For a first demo of the Open PHACTS pilot see http://www.youtube.com/OpenPHACTS.

Despite the technical challenges of connecting databases with completely different content and architecture, Open PHACTS faces also a substantial legal challenge. Every public data source is provided under a slightly different license. Although mostly based on Creative Commons, numerous slight modifications have been implemented by the different data provider. A platform connecting a set of data sources and allowing the public to query across all these sources needs to take also the legal issues into account. One possible solution might be that all data provider agree to a unified license model for the RDF version of their sources. This will not harm the original data source and allows setting up a large semantically enriched infrastructure, which can then be provided under a suitable open license model.

### 2.3 Introducing Standards to Assure Data Quality

As data repositories are growing at an exponential rate due to the huge amount of data coming from high-throughput screening initiatives, such as PubChem and EU-OPENSCREEN (http://www.eu-openscreen.de/), concerns about data quality and demands for setting standards are getting louder. Looking at integrated platforms for data retrieval, these issues are getting even more urgent to be addressed and finally solved.

Regarding quality of the data, we have to be aware that, for example, data from high throughput screens suffer usually from a high false positive rate and normally is validated through a series of confirmatory assays. Most often this is not captured in public data-sources and thus leaves users of the data exposed to assay artefacts and screening bias. Also compound structures may be depicted or named incorrectly in the sources and the high degree of cross-linking and circular references makes it very hard to ascertain compound provenance.^17^

In addition, as already mentioned before, there is also a strong need for unique identifiers. This is quite a difficult issue, as there is not a common agreement on when one thing equals to another. Is the identifier ‘gene sequence’ equal to ‘protein’? Additionally, synonyms are also widespread in the context of compound names, target names, or protein families. ABCB1, for instance, may be termed P-glycoprotein, P-gp, MDR1, and most probably some more.

Especially when integration of various data sources is performed, redundancy might present a nasty problem because it leads to very time consuming database cleaning processes.

On the other hand, giving a guarantee for data completeness will always stay a challenging and even unrealistic issue to fulfil in biology. For example, drug-target networks which serve to detect cross-pharmacology relationships among targets and to identify new targets for known drugs have to deal with this current limitation. As Vogt and Mestres pointed out, it happens that such public available drug-target interaction data is largely incomplete, and that the portion accessible is often inhomogeneous and biased towards targets of common therapeutic interest.^29^

Last but not least, the big diversity of available assays and screening results represents a tremendous problem as to the organisation, standardisation, integration, and analysis of the datasets. There have been efforts in order to address this problem, resulting in e.g. the first (beta) version of a free available webservice for semantic description of bioassays and HTS results, named BioAssay Ontology (BAO). The ontology is available online at the NCBO bioportal (http://bioportal.bioontology.org/ontologies/44531).^30^

In summary, setting standards is an important milestone one has to take in data integration processes. However, it should be stated that semantic web technology is only a tool that alleviates the data integration and is not designed for solving all the issues mentioned above. This will be the exercise of the whole community, and starts where biological/chemical data are generated and stored. Lately, the MIABE (Minimum information about a bioactive entity) guidelines were published, presenting a list of the items of information that should be provided when describing the synthesis and subsequent analysis of bioactive entities.^31^

### 2.4 A Versatile World of Chemical Compounds

Stepping aside the very complex and demanding process of data integration with all the challenges it possesses, the situation we are facing with respect to the amplitude of today’s chemical compound libraries itself is assigning further exercises to pharmaceutical R&D activities.

Analysing the distribution of the chemical features molecular weight (MW) and log*P*(o/w) (descriptors calculated with MOE) of the about 640 000 unique compounds in ChEMBL database revealed that around 490 000 compounds (76 %) fulfil the molecular weight (MW) criterion as stated in the rule of five (RO5).^32^ The same percentage of compounds does possess log*P* (o/w) values below 5. However, taking both important features into account around 63 % of all the unique compounds in ChEMBL fulfil these relevant criteria of drug-likeness. Thus, the possibility of using integrated data retrieval platforms synchronous with private *in house* data might present a prosperous strategy in the future.

As already mentioned in the section about standards that are needed when it comes to the integration of different databases, in the data exploitation process redundancy should be kept low on one hand, but most entire data (compound) collections are desired on the other hand. Thus – in addition to other identifiers – there is a need for unique canonical molecular identifiers for chemical compounds. However, the exact definition of compound uniqueness presents one of the major challenges in this huge world of chemical entities as it strongly depends on the type of chemical identifier used. Prominent examples for chemical notation systems are the SMILES strings,^33^ the CACTVS hash code^34^ and the new IUPAC InChIKeys (International Chemical Identifier).^35^ Hashed representations of such chemical graph identifiers do possess the additional advantage of masking the structural features of the molecules. Thus, they can be used for a secure data sharing process which is of very high priority when it comes to public-private partnerships.

Coming back to compound uniqueness, it should be stressed that the determination whether two or more compounds are unique or not always depends on the chemical characteristics we are looking for. How to take tautomerism, stereoisomerism, or salt composition into account will depend on the questions asked, and unfortunately to a large extent, how these issues are handled by the underlying source data. Recognising that this issue has in fact been solved many times in corporate chemical registration systems, EBI and the EBI Industry Programme recently hosted a workshop with contributions from chemical and pharmaceutical industry, informaticians and major public dataprovider (http://www.ebi.ac.uk/industry/Workshops/workshops.html). A similar initiative has been launched by FDA with a recently published guidance document on handling of chemical structures in databases and submissions (FDAs “*Food and Drug Administration Substance Registration System Standard Operating Procedure”,* available at http://1.usa.gov/snNNdn).

### 2.5 Case Study: Data Retrieval from ChEMBL for Human ABCB1 – Studying Bioassay Ontologies

In *academia* a major source for data retrieval in order to build up regression models, is the ChEMBL databank. It includes assay data of all different types available, including binding assays (measuring the interaction of the compound with the target directly), functional assays (often measuring indirect effects of the compound on a pathway, system or whole organism) and ADMET assays (measuring pharmacokinetic properties of the compound, interaction with key metabolic enzymes or toxic effects on cells/tissues). There has been an effort in order to standardize, where possible, the activity values to a preferred unit of measurement for a given activity type (e.g. IC50 values are displayed in nM, rather than µM/mM/M, half-life is reported in hours rather than minutes/days/weeks) which makes it easier to compare data across different assays.^17^

However, the problem of integrating activity measures coming from different assays persists. Basically, there are four different scenarios a researcher might face when searching for compound activities:

1. For one unique compound there might exist different activity values coming from different assays.
2. For one unique compound there might exist different activity values coming from the same kind of assay (but under slightly different conditions).
3. For one unique compound (reported to be targeting a certain protein) there might not be any activity value available which could be used for building up the desired model.
4. The data (activity values) do not unambiguously tell the user if the compound is a substrate or inhibitor of the target under investigation.

**Figure 1:**
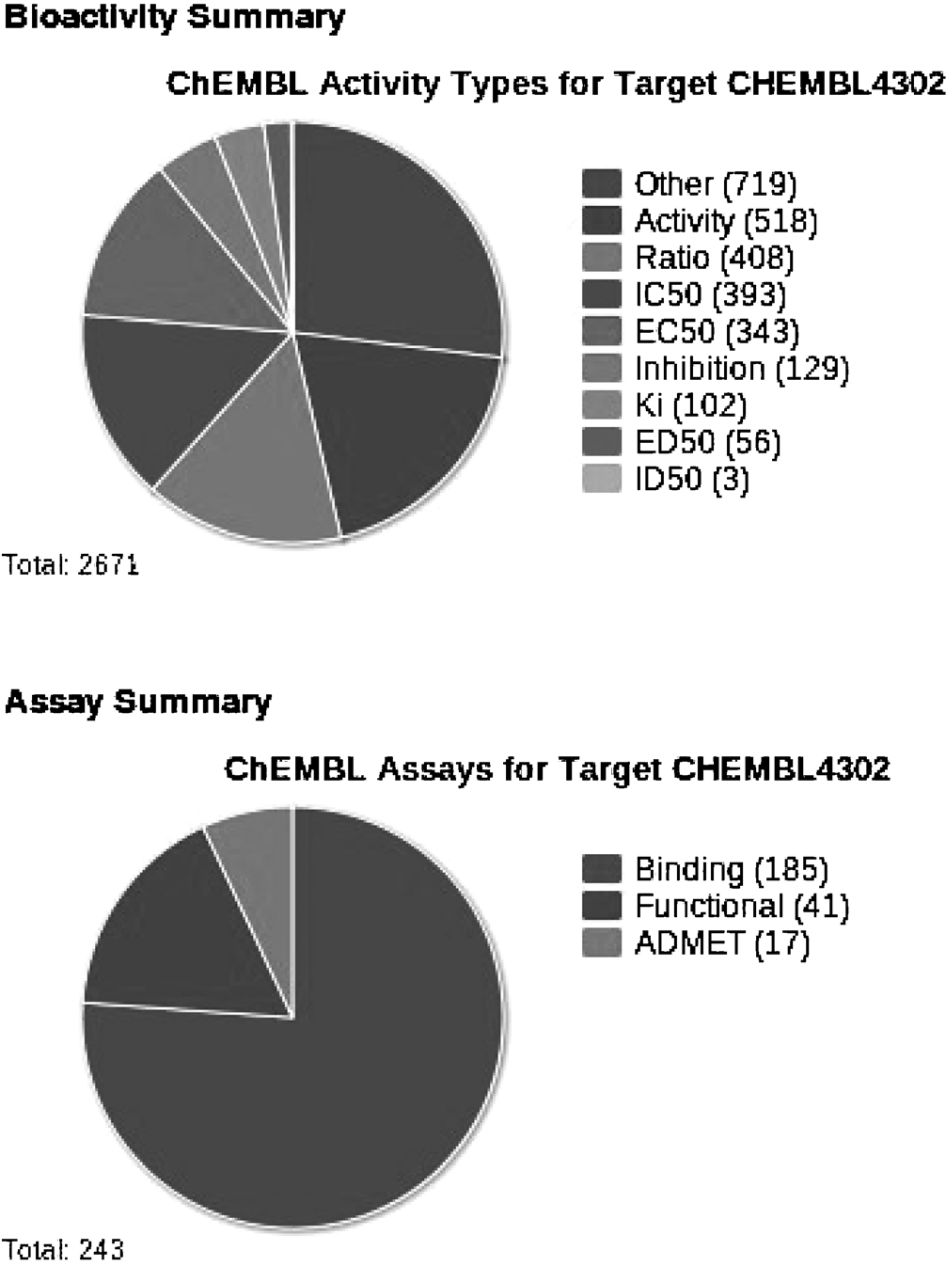
Bioactiovity and Assay Summary as depicted in ChEMBLdb for human ABCB1.

The use case we are describing in this section deals with human ABCB1 (human P-glycoprotein; CHEMBL4302). Figure 1 shows the different bioactivity types and different assays available for this transporter in ChEMBLdb.

**Figure 2:**
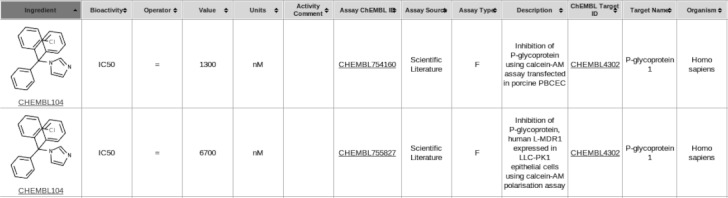
*IC*_50_ values for compound CHEMBL104.

As an example, Figure 1 shows that for ligands of human P-glycoprotein there are 393 IC_50_ activity values available. In some cases, there is more than one activity value for the same compound displayed. This is exemplified in Figure 2 for compound CHEMBL104.

In both cases the inhibition of human P-glycoprotein has been measured, but by the use of different bioassays. By the use of a ‘calcein-AM assay transfected in porcine PBCEC’ an IC_50_ value of 1300 nM was obtained. By also making use of a ‘calcein-AM polarisation assay’, but ‘expressed in LLC-PK1 epithelial cells’, the authors report an *IC*_50_ value of 6700 nm. This situation corresponds to scenario 1, as described before. Similar examples can be given for scenario 2, where the same assay produced different outcomes (data not shown).

Scenario 3 and 4 describe phenomena where the user’s knowledge is needed to decide whether a certain activity measure may serve as an input for model development. In scenario 3 it depends very much on the nature of the model that should be built (e.g. ‘Ratio’ values can only be used to build up binary models, i.e. linear or non-linear classification). Thus, the number of available compounds with activity values for the protein under investigation (e.g. in ChEMBLdb) may be somehow misleading, because not all the activity data can be used for model building.

Scenario 4 points towards a major issue in ABC transporter research. Deciding if a certain ligand of ABCB1 is either a substrate or an inhibitor might be a difficult task, depending on the kind of assay data available. Measures which display *IC*_50_ values, *K*_i_ values, or fall within the category ‘Inhibition’ in e.g. the ChEMBLdb ‘Bioactivity Summary’ (see Figure 1) should allow unequivocal assignment. However, categories like ‘Activity‘ or ‘Ratio’ (see Figure 1) might include both, substrates and inhibitors. In those cases, a compound series spanning a whole range of activity values might be classified into substrates or inhibitors according to certain thresholds which are defined on basis of expert knowledge. Finally, even more complicated, the pharmacological profile of a compound might change depending on the assay used. Thus, verapamil, cyclosporine A, and also propafenone analogs are inhibiting daunomycin efflux out of ABCB1 overexpressing tumour cells (i.e. act as inhibitors of ABCB1), but also act as substrates in polarized transport assays or ATPase activation assays. Thus, there is clear evidence that a P-gp inhibitor can at the same time also be a substrate.

The same is true for other targets. For instance, selective estrogen receptor modulators (SERMs) are reported to exhibit tissue-selective estrogen receptor (ER) agonist/antagonist properties.^36^ Thus, it is important that assays are able to encode the mode of action of the respective compound, which is, however, not always the case.

A deep knowledge about the outcome, strengths and limitations of each individual assay is therefore a basic requirement needed when performing a search of this kind. Thus, a sustainable infrastructure for complex data retrieval must certainly also include an appropriate bioassay ontology, alleviating researchers’ daily life.

## 3 Outlook

Being aware of the advantages that huge integrated data retrieval platforms will have for the drug-discovery community, stable, reliable, and sustainable web services of this kind – open to everyone – will increase in quantity and hopefully also in quality during the next years.

The latter may be achieved by addressing all the issues concerning quality control and setting standards – challenges that developers have to face now and in the near future.

Still, there are some kinds of data where there is only limited amount of public information available, thus it is a more delicate task to integrate such information. Data on ADMET properties of compounds, information on patents, and assay ontologies are only a few examples of pieces of information which are also needed to be integrated. Only by getting the most entire picture of the relationship between biological/pharmacological concepts, drug-discovery output will increase, which might finally also lead to better drugs.

## Biographical Information

*Barbara Zdrazil* studied pharmacy at the University of Vienna where she also received her PhD in medicinal chemistry in 2006. During her thesis she was predominantly working in the field of ABC-transporters, QSAR, and similarity-based descriptors. She received her postdoctoral training in the research group of Hans-Dieter Höltje at the Heinrich-Heine University of Düsseldorf. Currently, she is working in the Pharmacoinformatics Research Group at the University of Vienna, mainly involved in applying structure-based methods to study ligand recognition and transport by membrane transport proteins. She is also involved in the EU- projects eTOX and Open PHACTS.

*Niklas Blomberg* studied biochemistry at the University of Göteborg and did his PhD in biomolecular NMR at EMBL, Heidelberg in the group of Michael Nilges. He joined AstraZeneca in 1999 as computational chemist, in 2005 appointed team-leader for target-class and fragment based design and in 2006 became associate director for the Global Computational Chemistry and Cheminformatics Group. In 2011 he moved to AstraZeneca’s Respiratory & Inflammation research area to lead the Computational Chemistry/Computational Biology Team. His research interests include knowledge-driven approaches to target selection and early lead-generation. He is deputy industry chair for the Open PHACTS project.

*Gerhard Ecker* studied pharmacy at the University of Vienna, where he also received his PhD in medicinal chemistry in 1991. After his post-doctoral training at the group of J. Seydel in Borstel (Germany) he was appointed associate professor for medicinal chemistry at the University of Vienna in 1998 and full professor for pharmacoinformatics in 2011. He has published more than 100 articles related to SAR and QSAR studies on P-glycoprotein and his main scientific interests include pharmacoinformatic approaches to target drug transporters, in silico screening methods for promiscuous targets and antitargets, and non-linear methods in drug design. He is the academic coordinator of the Open PHACTS project and partner in the eTOX project.
